# Book Review

**Published:** 1926

**Authors:** 


### Reviews of Books.

---

**Diseases of the Heart.** By the late SIR JAMES MACKENZIE, F.R.S., M.D., F.R.C.P., etc. Pp. xxiv., 496. 4th edition, 1925. Oxford Medical Publications. Price 30s. To this, the last edition of the great book that made Mackenzie's world-wide fame, a pathetic interest attaches. It was written during the last years of his life, and—as he tells us in his preface—was planned as a re-statement of his views on cardiac disease. It is quite obvious, however, that this hope was not fully realised. The new work is there, and deeply interesting it is; but it has not been blended with the old as successfully as it would have been if Mackenzie's indomitable energy had been spared him a little longer. For the same reason there is a subacid flavour of criticism of all and sundry, which would doubtless have been diluted or mellowed if a longer consideration of the subject-matter had been possible. In spite of these defects, however, the book stands out, almost as strikingly as the first edition did, as the bold expression of a powerful and original mind. More than any other of his books it enshrines Mackenzie's personality, and constitutes a fitting monument to the memory of one who has set before his profession a splendid example of enthusiasm, honesty and devotion.

**Aphasia.** S. A. KINNIER WILSON, M.D., B.Sc. (Edin.), F.R.C.P. (Lond.). Psyche Miniature Medical Series. No. 2. Pp. 108. London: Kegan Paul. 1926. Price 2s. 6d. The approach to the subject of Aphasia may be from three avenues, the anatomical, the physiological and the psychological. All these have been attempted by various authorities, but no correlation of the three has so far been achieved. In this admirable résumé of the subject the contributions of each are reviewed. Lesions of an area of the left hemisphere comprising the posterior part of the frontal lobe, the island of Reil, the temporal lobe and the parieto-occipital area are apt to produce various forms of aphasia, anterior lesions being associated with loss of effector function and posterior lesions with loss of receptor functions. It is clear that the lesions in aphasia do not affect the actual efferent or afferent projector fibres and cells, since the use of the muscles and reception of sense impressions associated with other functions is unimpaired. Transcortical or association paths must therefore be concerned in the lesions. The degree of the representation of the speech function in the right hemisphere is discussed, and the most attractive theory would seem to be that speech is primarily represented bilaterally, but that the left hemisphere outstrips the right to a varying extent, and on this depends the possibility of speech recovery if the lesion of the left hemisphere is at all extensive and complete. The author rightly lays stress on the extreme complexity of speech as a psychological function, and while admitting the value of Head's recent contribution from the scientific standpoint, somewhat condemns it from the practical clinical standpoint. While this may be so, Wernicke's diagrams and the old motor and sensory aphasias will not do, and it remains for some future genius to correlate cerebral function with psychological "behaviour." Admittedly certain forms of aphasia are useful in localising cerebral lesions, but at the higher psychological levels, such as are involved in speech, the cortex acts as a whole and the discrimination of the different parts of the underlying mosaic of structure and function is for the future to determine.

**Mental Invalids: being the Morison Lectures delivered before the Royal College of Physicians of Edinburgh in June, 1925.** By C. C. Easterbrook, M.A., M.D., F.R.C.P., etc. Pp. 86. Edinburgh: Oliver and Boyd. Price 5s. This book contains a short series of interesting and instructive lectures by a practical man. The first contains a lucid explanation of the so-called New or Freudian Psychology. This will be welcomed by those who do not care to read the voluminous and involved literature on this subject; but though useful to have it explained in this way, it seems of doubtful good apparently to uphold a method which savours more of the old metaphysical discussions than of modern scientific research, and which, though undoubtedly a sound commercial proposition, is unscientific, irrational, and one fears, in many cases, unprofessional, and whose adherents are too often innocent of the anatomy, physiology and pathology of the central nervous system. Of the lecture on the clinical examination of mental invalids little need be said, except that though it might be called the counsel of perfection, it is very thorough, and after all the more information obtained the better, and it is only by endeavouring to gather a large amount that sufficient can be obtained. In the discussion of the causation of mental diseases perhaps too much stress has been laid on acquired psychosis. The question may be asked, "Would the psychosis have been acquired by an individual with a sound nervous system?" His histological classification of mental diseases is particularly happy, especially in Group B, in which, amongst other things, he separates the unfixed from the fixed delusional states, and both of these from the definitely organised and systematised delusional states of paranoia. The remarks on some aspects and methods of curative treatment will appeal to all who have the care of these unfortunate patients. He lays special stress on the need for early treatment in an institution, which at present is difficult in the case of the wealthy and impossible for the poor. His remarks on treatment by rest in bed in the open air are the more valuable as he points out that it is not the rest alone which is beneficial, but also the fresh air. In so many of our institutions too much insistence is placed upon the newly-admitted patient remaining in bed for a prolonged period in a ward, than which it would be difficult to imagine anything more depressing or enervating. He mentions hydrotherapy in one form or another as being useful, but does not labour the subject. Perhaps this is as well, for though it has a very high-sounding name, the treatment usually resolves itself into the continued administration of a warm bath for a prolonged period, frequently with a fair amount of mechanical restraint; it is a severe cardiac depressant, has been followed by a fatal termination in some cases, and would better be described as physical depression by legitimate means. These lectures will be read with great interest by those engaged in psychiatry, and may be confidently recommended to all who take an interest in the welfare of the insane.

**Migraine and other Common Neuroses.** By F. G. Crookshank, M.D., F.R.C.P. Psyche Miniatures, Medical Series, No. 1. Pp. 101. London: Kegan Paul. 1926. Price 2s. 6d. Two lectures are reprinted in this little volume, which demand the attention of every intelligent medical man, both from their inherent good sense and the charm of their style. The foundation of modern medicine on "Victorian Science and Mediæval Philosophy" is rightly deplored. The naturalistic approach which forbids the divorce of mind from body or body from mind in the study of disease, whether "functional or organic" is the method of choice, and the Adlerian doctrine of organ inferiority and its psychic compensation is in the author's view most fruitful in general psychotherapy. Migraine affords a good example of a neurosis which can be fruitfully attacked in this way. The organ inferiority of ocular defect and disharmony must be corrected. At the same time the psychical influence of the feeling of "rage and humiliation" in face of a situation which the patient cannot, or thinks he cannot, deal with successfully must be treated.

**Incapacity or Disablement in its Medical Aspects.** By E. M. Brockbank, M.B.E., M.D., F.R.C.P. Pp. viii., 120. London: H. K. Lewis & Co. Ltd. 1926. Price 7s. 6d. net. This little book is a veritable storehouse of facts and opinions on the medical aspects of disability arising out of a workman's employment. It is divided into two main sections, of which Section 1 deals with legal considerations and describes clearly all that a medical man need know about the various Acts connected with a workman's incapacity. Section 2 consists of notes, arranged in alphabetical order, of the commoner diseases and accidents connected with a workman's employment. Our only criticism of this book is that surgical conditions are dealt with far less fully than medical diseases. Whilst the balance between the two may be right for medical practitioners who require a reference book for questions arising out of certificates of incapacity under the National Health Insurance Act, and it is an excellent book for this purpose, as a guide for writing reports to insurance companies on cases of accidents it is a little weak on its surgical side. For example, fractures are not mentioned as a cause of disability, nor osteo-arthritis following an accident, and these are two of the most common causes of incapacity with a legal side. Traumatic neurasthenia, admittedly a very vexed subject, is not at all clearly described; this is much to be regretted, as this subject is becoming of increasing importance in County Courts, and definite teaching would have been of much value. On the other hand, the author deserves credit for the very excellent pages on cardiac disability which is dealt with from many aspects. The notes on blood-pressure, pneumonia and tuberculosis are also worthy of special notice. This book is throughout well written, and there is a welcome absence of legal terminology and long sentences that make most books dealing with legal subjects almost unintelligible to a medical man. It will be found a most useful little volume for medical men called upon to deal with the many questions relating to incapacity from employment.

**Physical Fitness in Middle Life.** By F. A. Hornibrook, with Foreword by Leonard Williams, M.D. Pp. vi., 116. London: Cassell & Co. 1925. Price 6s. net. This book, so easy to read and understand, gives in a brief, concise manner the author's experience of how to maintain physical fitness in that period of middle life when so many men run to flesh. He points out how the ever-increasing addition to a man's weight acts in a vicious circle by making him disinclined to take any exercise beneficial to the lower half of his trunk. This neglect shows itself in stagnation of the bowels, with absorption of toxin material as evidenced by short temper, midday drowsiness, etc. He illustrates in a very clever manner how modern athletics tend to develop just one set of muscles to the detriment of good health generally by quoting the case of a cycle sprinter whose physique, he says, is more often a specimen fit for a pathological laboratory than a model for artists. In one chapter he traces how civilised man is taught to curb his natural functions of evacuation from the days of the cradle till he arrives at man's estate, when he is one of the applicants for attention from a firm of English druggists who claim to sell 70,000,000 tablets of cascara a year. This admiration for the results obtained by the physical exercises and devices of the uncivilised nations in producing such perfect forms of manhood is worthy of our attention. This most valuable addition to our knowledge closes with a few very practical hints as to how to work well, sleep well and feel well.

**An Introduction to Surgery.** By Rutherford Morison, M.D., F.R.C.S. (Edin.), Emeritus Professor of Surgery, Durham University, and Charles Saint, C.B.E., M.D., M.S., Professor of Surgery, Cape Town University. Second edition. Pp. 347, figures 183. Bristol: John Wright & Sons Ltd. 1925. Price 15s. Both as a surgeon and as a teacher Professor Morison is so eminent that his writings carry very great weight. In the case of the present volume the second edition has been compiled with the assistance of Professor Saint, who was Morison's pupil. Its method is original and its matter full of practical interest. It aims at teaching general principles, with a view to training the student to think out surgical problems for himself. We are not favourably impressed by the method of arrangement. This, no doubt, has a strict basis of reason founded on structure and function, but we think that it is likely to confuse rather than to help a beginner. For example, the hollow muscular systems, stomach, intestines, ureters, bladder and uterus, are considered together, but one looks in vain for a simple description of acute intestinal obstruction or a strangulated hernia. Then again, undue prominence is given to matters about which opinions may differ and to matters of chiefly theoretical interest, *e.g.* the treatment of psoas abscess by excision or the rôle of the great omentum and the natural cure of diseases. But in spite of these drawbacks the book is of great interest and every paragraph is of importance. The teaching value of the book is greatly enhanced by the beautifully clear photographs and line drawings.

**Operative Orthopædics.** By A. STEINDLER, M.D., F.A.C.S., Professor of Orthopædic Surgery, Iowa. Pp. 403, figures 83. D. Appleton & Co. 1925. Price 30s. In some respects this is a valuable addition to the text books of orthopædic surgery. It is well and clearly written, and many of the diagrammatic illustrations are excellent. The aim of the author is to present each operative procedure in four aspects, its anatomical and physiological rationale, the clinical possibilities, the operative technique and the statistical results. But this aim is not very fully carried out except in the earlier chapters. Chapters on the simple deformities and tendon transplantation are clear and helpful, but those on such subjects as bone grafting or kineplastic amputations are much too brief to be of any practical value. In subsequent editions we think the author will have to choose between making the book smaller by confining it to the treatment of deformities or making it larger by dealing more fully with the major problems of bone and joint surgery.

**The Treatment of Fractures and Dislocations in General Practice.** By C. MAX PAGE, D.S.O., M.S., F.R.C.S., and W. ROWLEY BRISTOW, M.B., B.S., F.R.C.S. Second edition. Pp. 279. Illustrated. London: Henry Frowde and Hodder & Stoughton 1925. Price 12s. 6d. net. The frequency with which bony injuries figure in general and hospital practice calls for familiarity with diagnosis and treatment on the practitioner's part. To this end no clearer nor more concise compendium can be consulted than this handy volume with its admirable illustrations. It is a fine product of attractive exposition and modern handling of fractures, missing nothing from head to foot and never failing to give sound, simple advice on treatment. Happily the neural complications of elbow injuries receive due mention in this larger second edition (forty pages longer), and the inclusion of notes on dislocations is a welcome addition. One hesitates to offer anything but high praise for such a reliable and up-to-date treatise, yet one feels the method of dealing with the fractured femur advocated recently by Mr. Hamilton Russell deserves recognition.

**Rational Gland Therapy for Women.** By I. WANLESS DICKSON, M.B., F.R.C.S. Pp. viii., 96. London: H. K. Lewis & Co. Ltd. 1926. Price 4s. 6d. The writer of this book is an enthusiast in the treatment of diseases by ductless gland preparations, and there is much of interest in his little monograph. But he is hardly justified in being as dogmatic as he is in many of his statements with regard to the use of such therapy in gynæcology and obstetrics, while the whole subject of endocrinology and its application to practical medicine is still so much a matter of theory and experiment. He strongly advocates the value of mammary gland substance in the treatment of menorrhagia and dysmenorrhœa, while hardly giving sufficient credit to the good results obtained by the administration of thyroid gland preparations for these symptoms, nor does he refer to the value of the latter in the treatment of primary and secondary sterility. The vomiting of pregnancy, pernicious and other, is referred to instability of the pituitary gland, without in any way taking into consideration the modern views on the production of the toxæmias of pregnancy. It is unlikely that gynæcological surgeons of experience will agree with his statement that "the nearer a woman approaches the menopause the less is she able to withstand the shock of a surgically produced menopause, particularly if the ovaries are removed at the same time." It is more reasonable to expect that the shock and disturbance will be most severe when an artificial menopause is brought on during the period of greatest sexual activity. The writer attaches very serious risks to the administration of ovarian substances in the treatment of menopausal symptoms. These, in the opinion of the reviewer, have been very much over-estimated.

**An Introduction to the Study of X-rays and Radium.** By HECTOR A. COLWELL, M.B. (Lond.), and CECIL P. G. WAKELY, F.R.C.S. (Eng.). Pp. x., 203. 37 illustrations. Oxford Medical Publications. 1926. Price 10s. 6d. This is a too short, neatly dovetailed account (by two practical teachers) of X-ray and radium and other radio-active agents from the scientific point of view, with special reference to the actinic potency of X-rays, to the protection necessary for operator and patient against these agents, to their action on healthy living tissues and undiseased growth (with the object of palliation and even possible cure of the latter). The book challenges comparison with Kaye's work on "X-rays" (1914), in reference to the more recent developments of the atomic theory. It contains some interesting tables and anatomical landmarks. This book is refreshing in that it has no X-ray picture in it, nor question-begging clinical cases, nor unconvincing statistics of cure. I hope soon to see further and enlarged editions of this little book. To the statement on page 26 that X-ray pictures are *not* "images" I must demur. If the authors will carefully take a pinhole photograph and then a radiograph of such an object as an articulated skeleton hand and forearm (from the same point of sight and from the same aspect of the object), adding thereto an encircling metallic bracelet and one or two finger rings, they will have some difficulty in making up their minds as to which is the best "image" of the two pictorially. Perhaps some skilled draughtsman could make them wise as to the real essential peculiarities of these two similar representations, the one made by reflection of light the other by interception of X-rays. As usual, the word "Perspective" is not to be found in index or text. There is no recognition that the advantages claimed for an "orthodiagram" may be more simply and inexpensively obtained by divergent X-rays at ordinary distances comparable with the length of one's arm. There is no mention of the mercury vapour lamp as a rectifier or converter of an alternating current supply. There is no allusion to the small sesamoid bones in the tendons of the hand and foot or those occasionally found in other parts. As usual, also, it is tacitly assumed that the fluorescent screen is not merely a translucent window blind (when lighted up, viewed from the street), but to be some sort of transparent window pane. This book marks a distinct advance in medical X-ray literature.

**Treatment of Gonococcal Infection by Diathermy (with an Appendix on the Treatment of other forms of Arthritis by Diathermy).** By E. P. Cumberbatch, M.A., B.M., B.Ch, (Oxon.), M.R.C.P., and C. A. Robinson, M.B., B.Ch., D.M.R.E. (Cantab.). Pp. 150. London: Heinemann Ltd. 1925. Price 7s. 6d. net.—These pioneers of diathermy have provided a useful book for those interested in the treatment of gonococcal infection of certain regions, such as the prostate in males, the cervix in females, and joints which hitherto have proved most resistant to the usual routine treatment. They have secured wonderful results where other methods, as irrigation, vaccines and external application, have been a dismal failure. It is a book which from its conveniently small size might well be called a pocket book, and will repay any practitioner or student for its perusal.

**The Basis of Vital Activity.** By Sir James Mackenzie, M.D., F.R.S., F.R.C.P., etc., with a Foreword and a short biographical sketch ; and a note by James Orr, M.B. Pp. 132. London : Faber and Gwyer. 1926. Price 6s. net.—This brief summary of five years' work at the St. Andrew's Institute for clinical research presents, in a conveniently compact form, observations and reflections which are scattered through the last edition of Sir James Mackenzie's book on *Diseases of the Heart*. The Foreword tells us that "less than forty-eight hours after the last sentences of this manuscript were written Sir James Mackenzie succumbed," by a strange irony, to disease of a kind that he had spent his life in studying. The book is very short and lucid, and intensely stimulating. Every doctor ought to read it.

**A Text Book of the Practice of Medicine.** By various authors. Edited by Frederick W. Price, M.D., F.R.S. (Edin.). Pp. xxxvi., 1,828. London: Humphry Milford (Oxford University Press). 1926. Price 35s.—Since its first appearance, less than four years ago, four additional impressions of this volume have been printed. The editor has wisely decided to issue a fresh edition, which includes new articles on such subjects as Tularæmia, Botulism, Apical Dental Infection, Chylous Diarrhœa, and so forth ; as well as additional information on many topics. We were much impressed by the first edition, and can only repeat the favourable opinion offered then, and confirmed by the subsequent success of the book. We know of no better summary of the British point of view in medicine than that which is presented in this compact volume.

**What to do in Cases of Poisoning.** By WM. MURRELL, M.D., F.R.C.P., revised by P. HAMILL, M.D., D.Sc., F.R.C.P. (Lond.). Ed. 13, pp. 276. London: H. K. Lewis & Co. Ltd. 1925. Price 4s. 6d. net.—This little book might well be described as *multum in parvo*, and despite its size, seems to be quite an exhaustive treatment of its subject matter. The antidote section is particularly well tabulated, and the careful preservation of specimens in view of subsequent inquiry is very properly emphasised. The medical practitioner's bookcase is sometimes in danger of being over-stocked, but this small volume should certainly find a place on it.

**The Gastric Function in Health and Disease.** By JOHN A. RYLE, M.D. (Lond.), F.R.C.P. Pp. viii., 152. Oxford Medical Publications. 1926. Price 8s. 6d. net.—In this short monograph Dr. Ryle discusses the recent advances which have been made in our knowledge of gastric function in health and disease, more especially as demonstrated by the employment of the bismuth meal and the fractional test meal. These methods have completely revolutionised our views as to the meaning of the subjective and objective symptoms of dyspepsia, and all practitioners of medicine must be prepared to sacrifice their old theories in the light of modern research. The author, like others of the Guy's School, places great emphasis on the part played by the stomach muscle tonus and by the exaggeration or depression of normal reflexes. The book is full of interest, and will be read with advantage both by the student and the practitioner. We would especially recommend the chapters on the classification of the dyspepsias and that dealing with gastrojejunostomy for duodenal ulcer. In the latter Dr. Ryle lays down some general rules for assessing the suitability of cases for operation and considers the treatment of post-operative dyspepsias.

**Diseases of the Nervous System.** By H. CAMPBELL THOMSON, M.D., F.R.C.P. (Lond.), and GEORGE RIDDOCH, M.D. (Aber.), F.R.C.P. (Lond.) Fourth edition. Pp. xvii., 541. London: Cassell & Co. 1925. Price 16s. net.—The revised fourth edition of this book maintains the high level of its predecessors. It contains an enormous amount of information clearly and concisely put. The subject-matter is well arranged and carefully indexed. There is a wealth of first-class illustrations, largely instantaneous photographs. In addition there are a number of beautifully-coloured plates, extremely well produced. Section VIII., dealing with psychoneuroses is particularly well done, and seems to contain the essence of modern teaching with regard to these conditions, without any unnecessary detail or irrelevant matter. Where all is so good it is difficult to criticise. One or two small omissions may be noted. In the section on Disseminated Sclerosis no mention is made of the loss of superficial reflexes. In describing peripheral polyneuritis no stress is laid on the symmetrical character of the condition. No attempt is made to distinguish between neurasthenia proper and the anxiety neuroses. The book may be strongly recommended as one of the best smaller text-books we have on nervous diseases.

**An Introduction to Forensic Medicine.** By H. A. Burridge, M.A., M.B. (Dublin). Pp. xiii., 455. London: H. K. Lewis & Co. Ltd. 1924. Price 10s. 6d. net.—Dr. Burridge's venture into the realm of medico-legal literature is quite a good effort, and his manual can be described as a successful compromise between a synopsis and a text-book. Founded, as he states, on his lectures, it well covers the syllabus laid down by the examining bodies, and the scope of its contents is ample for a pass. For this reason we commend it to students as well as practitioners who may like to have a moderately priced text-book at hand for reference.

